# Audiological characteristics of children with congenital unilateral hearing loss: insights into Age of reliable behavioural audiogram acquisition and change of hearing loss

**DOI:** 10.3389/fped.2023.1279673

**Published:** 2023-11-08

**Authors:** Vicky W. Zhang, Sanna Hou, Angela Wong, Christopher Flynn, Jane Oliver, Michelle Weiss, Stacey Milner, Teresa Y. C. Ching

**Affiliations:** ^1^ Audiological Science Department, National Acoustic Laboratories, Sydney, NSW, Australia; ^2^ Department of Linguistics, Macquarie University, Sydney, NSW, Australia; ^3^ Lutwyche centre, Hearing Australia, Brisbane, QLD, Australia; ^4^ Upper Mt Gravatt centre, Hearing Australia, Brisbane, QLD, Australia; ^5^ Dandenong centre, Hearing Australia, Melbourne, VIC, Australia; ^6^ Cheltenham centre, Hearing Australia, Melbourne, VIC, Australia; ^7^ NextSense Institute, Macquarie Park, Sydney, NSW, Australia; ^8^ Macquarie School of Education, Macquarie University, Sydney, NSW, Australia; ^9^ School of Health and Rehabilitation Sciences, University of Queensland, St Lucia, QLD, Australia

**Keywords:** unilateral hearing loss, children, audiological characteristics, behavioural audiogram, progressive hearing loss, etiology

## Abstract

**Objectives:**

The aims of this study were to report the audiological characteristics of children with congenital unilateral hearing loss (UHL), examine the age at which the first reliable behavioural audiograms can be obtained, and investigate hearing changes from diagnosis at birth to the first reliable behavioural audiogram.

**Method:**

This study included a sample of 91 children who were diagnosed with UHL via newborn hearing screening and had reliable behavioural audiograms before 7 years of age. Information about diagnosis, audiological characteristics and etiology were extracted from clinical reports. Regression analysis was used to explore the potential reasons influencing the age at which first reliable behavioural audiograms were obtained. Correlation and ANOVA analyses were conducted to examine changes in hearing at octave frequencies between 0.5 and 4 kHz. The proportions of hearing loss change, as well as the clinical characteristics of children with and without progressive hearing loss, were described according to two adopted definitions: Definition 1: criterion (1): a decrease in 10 dB or greater at two or more adjacent frequencies between 0.5 and 4 kHz, or criterion (2): a decrease in 15 dB or greater at one octave frequency in the same frequency range. Definition 2: a change of ≥20 dB in the average of pure-tone thresholds at 0.5, 1, and 2 kHz.

**Results:**

The study revealed that 48 children (52.7% of the sample of 91 children) had their first reliable behavioural audiogram by 3 years of age. The mean age at the first reliable behavioural audiogram was 3.0 years (SD 1.4; IQR: 1.8, 4.1). We found a significant association between children's behaviour and the presence or absence of ongoing middle ear issues in relation to the delay in obtaining a reliable behavioural audiogram. When comparing the hearing thresholds at diagnosis with the first reliable behavioural audiogram across different frequencies, it was observed that the majority of children experienced deterioration rather than improvement in the initial impaired ear at each frequency. Notably, there were more instances of hearing changes (either deterioration or improvement), in the 500 Hz and 1,000 Hz frequency ranges compared to the 2,000 Hz and 4,000 Hz ranges. Seventy-eight percent (*n* = 71) of children had hearing deterioration between the diagnosis and the first behavioural audiogram at one or more frequencies between 0.5 and 4 kHz, with a high proportion of them (52 out of the 71, 73.2%) developing severe to profound hearing loss. When using the averaged three frequency thresholds (i.e., definition 2), only 26.4% of children (*n* = 24) in the sample were identified as having hearing deterioration. Applying definition 2 therefore underestimates the proportion of children that experienced hearing changes. The study also reported diverse characteristics of children with or without hearing deterioration.

**Conclusion:**

The finding that 78% of children diagnosed with UHL at birth had a decrease in hearing loss between the hearing levels at first diagnosis and their first behavioural audiogram highlights the importance of monitoring hearing threshold levels after diagnosis, so that appropriate intervention can be implemented in a timely manner. For clinical management, deterioration of 15 dB at one or more frequencies that does not recover warrants action.

## 1. Introduction

Universal Newborn Hearing Screening (UNHS) programs have played a crucial role in identifying unilateral hearing loss (UHL) in infancy. Since its implementation, there has been a significant increase in the prevalence of UHL in newborns, rising from approximately 0.3–0.6 per 1,000 (1, 2) to 1–2.4 per 1,000 children (3–6). This increase has drawn increased attention to the impact of UHL during early childhood. Previous studies have shed some light on the significant impacts of UHL on various aspects of development in certain children. These impacts include difficulties in sound localization, speech recognition in noise (7–13), and higher-level language skills such as cognition, comprehension, reading, and communication (12, 14). Long-term impacts have also been reported, indicating that children with hearing loss may experience poorer outcomes such as overall quality of life (15), academic achievements, and psychosocial challenges, compared to children with normal hearing in both ears (16). The early identification of UHL is essential to ensure timely and effective intervention for optimal developmental outcomes. However, clinical management of UHL still presents several challenges due to limited knowledge regarding the audiological characteristics, underlying causes, and a lack of evidence-based information regarding the long-term consequences in this target population.

The accurate diagnosis and timely intervention as early as possible are widely recognized as crucial for optimal audiological management of hearing loss configuration, progression, and long-term outcomes in children with hearing loss (17, 18). Currently, electrophysiological tests such as auditory brainstem response (ABR) and auditory steady-state response (ASSRs) measures are commonly used to estimate children's behavioural audiograms. It should note that these tests only provide approximations. For instance, tone-burst evoked auditory brainstem response (TBABRs) has an error range of ±5–20 dB when determining behavioural results at different frequencies (19–21). Additionally, there is no reliable method to estimate hearing threshold for the impaired ear of a child with unilateral auditory neuropathy spectrum disorder until they can provide reliable individual ear behavioural testing. These highlight the need for a reliable behavioural audiogram for each ear. However, acquiring ear-specific reliable behavioural audiograms can be challenging, especially for children with UHL, as they must be able to accept the use of insert-phones or headphones during the testing. Furthermore, due to the asymmetrical hearing levels, masking is often required, which also adds to the complexity during behavioural testing. The challenge is further exacerbated by some other non-technical factors such as the child's developmental stage, general cooperation, and limited time allocated for clinical appointments. There is limited information available regarding the average age at which reliable behavioural hearing levels can be obtained in children with congenital UHL diagnosed via UNHS.

Another challenge lies in understanding the prevalence rate of progressive hearing loss in children with UHL, and its potential risk factors. Despite the fact that children with UHL can still perceive sound through their unaffected ear, the importance of obtaining reliable behavioural audiograms and regularly monitoring hearing thresholds for each ear may have been insufficiently emphasized. Studies have indicated that a considerable proportion of children with UHL are at risk of progressive hearing loss, either in one or both ears (22, 23). However, the percentage of children with UHL who exhibit hearing deterioration varies across reports. This may be due to differences in definitions of progressive hearing loss used, age ranges of the children, measurement methods, follow-up durations and specific sub-groups of hearing loss under investigation (22, 24–30). For example, Dahl and colleagues (25) defined progressive hearing loss as a decrease in 10 dB or greater at two or more adjacent frequencies between 0.5 and 4 kHz or a decrease in 15 dB at one octave frequency in the same frequency range over the period of investigation. Studies on UHL that adopted this definition have reported progressive hearing loss in 37%–47.5% of children with UHL, with 11.9%–19% eventually developing bilateral hearing loss (22, 23, 26). The figures are not directly comparable, which may be due to the different sample sizes and variations in baseline and the most recent audiometric assessment points used in the studies. Several other studies have also used averaged hearing thresholds from three or four frequencies, considering hearing changes of ≥10 (31), ≥15 (32), or ≥20 dB (16) as indicative of progressive hearing loss in children with UHL. For instance, according to the Fitzpatrick study (16), 12.9% of children with UHL were reported to have a change of ≥20 dB in the pure-tone average of three frequency thresholds from 0.5 to 2 kHz. In comparison, Purcell et al. (31) found that 32.8% of children with UHL, specifically associated with ipsilateral bony cochlear nerve canal stenosis, experienced progressive hearing loss, defined as a change of ≥10 dB in pure-tone thresholds averaged across 0.5, 1 and 2 kHz. The study started hearing assessments from a mean age of 7.7 years and followed-up participants for approximately 3 years (1,126 days). Additionally, Paul et al. (33) reported a rate of 19% for progressive hearing loss when using a similar criterion of >10 dB change in the pure-tone average of four frequency thresholds from 0.5 to 4 kHz. In terms of measurement points and lengths of time between measures, different studies have focused on various approaches. For example, Fitzpatrick et al. (23) compared the diagnostic audiogram (median age 3.3 months) to the most recent audiometric assessment (median age 88.8 months) with a median length of 64.3 months between measurements. They found that 47.5% of the children showed progressive loss, and 11.9% progressed to bilateral loss. Another study by Fitzpatrick et al. (34) reported that 8 out of 62 children with UHL showed progressive hearing loss (12.9%). The figure, based on data collected from 1990 to 2010, is lower than the 2023 report, which could be due to some children in the 2014 study not being identified with hearing loss until after 5 years of age (i.e., after the implementation of a UNHS program). Moreover, the potential factors contributing to progressive hearing loss in UHL children still remain inconclusive (35). Some studies suggested that cytomegalovirus (CMV) (36–38) and mutations in the gap junction *β* 2 gene (GJB2) (39) are risk factors. However, other studies have not found significant associations between progressive hearing loss and indicators such as genetic mutations and, NICU admission, family history, craniofacial anomalies, syndromes, postnatal infections (22, 25), or factors like age at diagnosis, severity of hearing loss, or etiologic (23, 26).

Due to the challenges associated with obtaining a reliable behavioural audiogram during early childhood, and the variations in reported proportions and potential risks of progressive hearing loss documented in different studies on UHL, there is a need for more evidence to determine the age at which a reliable audiogram can be obtained, as well as to gather more information on the extent of hearing changes in children with UHL. This knowledge will contribute to the development of optimal management and intervention strategies. Considering these challenges and the importance of early intervention, this study aims (1) to describe the audiological characteristics and etiology of a group of children diagnosed with congenital unilateral hearing loss (CUHL); (2) to examine the age at which reliable behavioural audiograms can be obtained and explore potential factors that could delay obtaining these audiograms; and (3) to investigate changes in hearing levels from birth to the first reliable behavioural audiogram, as well as the potential risks of progressive hearing loss.

## 2. Materials and methods

### 2.1. Participants

Participants included 91 children who were enrolled in the Children with Unilateral Hearing Loss study in New South Wales (*n* = 28), Victoria (*n* = 24), and Queensland (*n* = 39). All participants were identified with UHL via newborn hearing screening with a subsequent diagnosis of permanent UHL confirmed via electrophysiological hearing tests at diagnostic centres and hospitals. These include tympanometry, distortion product otoacoustic emission (DPOAEs), and TBABRs or ASSRs testing on both ears at a diagnostic centre or hospital for birth dates between March 19, 2014, and February 8, 2018. Following diagnosis of the hearing loss, children were referred to Hearing Australia (the national government funded organisation that provides hearing services to all children with hearing loss under the age of 26 years in Australia) to receive further audiological services, which includes ongoing hearing assessments, hearing device fitting and verification. Inclusion criteria for this report included children enrolled in the study on unilateral hearing loss (reported separately) who had (1) a diagnosis of UHL; (2) frequency-specific audiometric thresholds estimated from electrophysiological testing at diagnosis; (3) reliable behavioural audiograms in early childhood at least at one low frequency (0.5 or 1 kHz) and one high frequency (2 or 4 kHz) at Hearing Australia (see details in Section 2.2, “Audiological data collection for the first reliable behavioural audiogram”). Additionally, children diagnosed with Auditory Neuropathy Spectrum Disorder (ANSD) were excluded from the sample due to the inaccuracies in estimated hearing thresholds using electrophysiological hearing tests at the time of diagnosis. After a comprehensive review of records at Hearing Australia, data of children who met the inclusion criteria were included in this report. This study has been approved by Hearing Australia Human Research Ethics Committee (No. AHHREC2014-28 and No. AHHREC2019-9).

### 2.2. Data collection procedures

#### 2.2.1. Audiological data collection at diagnosis

Diagnostic data for this study were collected at the time when children were diagnosed with UHL. The hearing thresholds at diagnosis in each ear were measured by audiologists at diagnostic centres or hospitals using objective electrophysiological tests of TBABRs or ASSRs. The correction factors applied to convert the electrophysiological hearing results into estimated behavioural hearing threshold (dB eHL) were as follows: for TBABRs, 500 Hz was adjusted by 10 dB, 1,000 Hz by 10 dB, 2,000 Hz by 5 dB, and 4,000 Hz by 0 dB; for ASSRs, 500 Hz was adjusted by 15 dB, 1,000 Hz by 10 dB, 2,000 Hz by 10 dB, and 4,000 Hz by 10 dB (19, 40, 41). The age at diagnosis was defined as the age of hearing loss confirmed at the diagnostic centre or hospital using relevant electrophysiological methods.

#### 2.2.2. Audiological data collection for the first reliable behavioural audiogram

The behavioural audiological assessments after diagnosis for all children were performed by clinical paediatric audiologists at Hearing Australia according to the national audiological protocols. The behavioural hearing thresholds were obtained using visual reinforcement audiometry (VROA), conditioned play audiometry (PA), or a combination behavioural method of VROA and PA if needed, depending on the child's age and ability. Serial behavioural audiological results after the time of diagnosis for each child were retrospectively reviewed by experienced research audiologists from clinical records held at Hearing Australia, to identify reliable behavioural audiograms. The definition of a reliable behavioural audiogram for the impaired ear includes:

• clinical note shows reliable behavioural results on the tested frequencies.
• must have hearing thresholds at least at one low frequency (0.5 or 1 kHz) and one high frequency (2 or 4 kHz).
• must have masked hearing thresholds when required, for air and bone conduction thresholds.

Specific decision rules were developed for this study, such as if an audiogram associated with temporary abnormal middle ear function, it was not recorded as a reliable result, and subsequent audiological results were examined accordingly. The date and the detailed audiological results (e.g., hearing thresholds, transducer, assessment method, clinical comments) of the first reliable behavioural audiogram were then recorded for further analysis.

#### 2.2.3. Factors affecting the age of obtaining reliable behavioural audiograms

To identify potential factors affecting the age at which reliable behavioural audiograms were obtained, children's demographic and basic audiological information, as well as the etiological details were extracted from available clinical reports of Hearing Australia and provided reports by other diagnostic organizations to Hearing Australia. To gain further insights of clinicians' experiences and challenges on the potential reasons that may affect how early a behavioural audiogram is obtained, we invited clinical audiologists at Hearing Australia to complete an informal online survey^1^. According to the survey results and content analysis of additional comments provided by audiologists, a few other reasons were identified, apart from the collected children's demographic data, audiological characteristics and etiological details. These additional factors included children's behaviour/compliance, staff and equipment resources, scheduled appointments time, as well as caregivers' attitudes toward UHL and family availability.

Considering the suggested reasons from the survey, we reviewed the clinical case notes of earlier testing appointments before obtaining the first reliable behavioural audiogram. We then grouped the comments reported during the actual appointments into three categorised reasons: (1) children's behavioural issues (such as loss of interest in tasks, attention issues, or intolerance of earphones), (2) challenges unrelated to children's behavioural issues (such as insufficient masking information for children requiring masking, or insufficient appointment time for masking), and (3) family availability (such as lost contact or failure to attend scheduled appointments). Cases exhibiting any of these issues were labelled as “Yes”, while those without such issues were labelled as “No”. These three potential reasons and the other four clinical variables (presence or absence of reported etiology, degree of hearing loss at diagnosis, ongoing middle ear problems in the impaired ear, and hearing device fitting) were included in subsequent statistical analysis and discussion on the reasons of the delay in obtaining behavioural audiogram in this report.

#### 2.2.4. Definition of degree of hearing loss

The degree of hearing loss in the impaired ear at diagnosis and behavioural audiological assessments was further determined by three-frequency averaged thresholds at 0.5, 1, and 2 kHz (22, 26). All children included in this report had all three frequency-specific audiometric thresholds (0.5, 1, and 2 kHz) estimated from electrophysiological testing at diagnosis in each ear. For the behavioural audiograms in the impaired ear, all children had measured thresholds at 1 kHz, whilst 15 children had missing data at 0.5 kHz, 19 children had missing data at 2 kHz. To address this, a general rule using an estimation method by extrapolating from the available measured frequency-specific results (as described in (42) was used to make the best estimate of behavioural hearing thresholds for the missing values:

• If there is no measured threshold at 0.5 kHz, extrapolate the results by decreasing 10 dB from 1 kHz;
• The missing threshold at 2 kHz is calculated as the mid value between the 1 and 4 kHz;

The three frequencies averaged hearing level (3FAHL) in the impaired ear was then calculated using the thresholds at 0.5, 1, and 2 kHz.

#### 2.2.5. Definition of changes in hearing level

For the purposes of this study, the changes in hearing were determined by comparing the hearing thresholds measured from the first reliable behavioural audiogram to the baseline hearing thresholds at diagnosis. A positive difference between two thresholds indicated a deterioration in hearing, while a negative difference indicated an improvement. To be consistent with recent literature, the following two definitions were adopted for the analysis of significant deterioration:

• Definition 1: a decrease of ≥10 dB at two or more adjacent frequencies between 500 and 4,000 Hz, or a decrease of >15 dB at one octave frequency in the same frequency range (22, 23, 25, 26).
• Definition 2: a change of ≥20 dB in the three frequencies (500, 1,000, and 2,000 Hz) pure-tone average (16, 22, 28).

Additionally, this study employed a similar version of definition 2 to determine a significant improvement in hearing thresholds. Specifically, an increase of greater than or equal to 20 dB (i.e., a change of ≤−20 dB) in the three-frequency average hearing level (3FA HL) was defined as an improvement. Any change that did not meet the criteria for deterioration or improvement (i.e., fell within the range of −20–20 dB in 3FA HL) was categorized as stable hearing loss between the estimated baseline hearing threshold and the first reliable behavioural audiogram.

### 2.3. Statistical analysis

The main interests of analysis in this study were the age at the first reliable behavioural audiograms and the proportion of children with change of hearing threshold levels. Descriptive statistics, such as mean and standard deviation, median and percentiles, interquartile range were used to report quantitative outcomes. Regression analyses were used to examine relationships between potential factors and the ages at which reliable behavioural audiograms were obtained. The potential reasons for delayed behavioural audiograms in our sample were extracted from clinical notes and classified based on a survey's results (see the footnote in Section 3.2). Pearson's correlation analysis was used to assess the amount of change in hearing thresholds at individual frequencies (0.5–4 kHz) between the baseline hearing thresholds at diagnosis and the hearing thresholds measured from the first reliable behavioural audiogram. A two-way ANOVA test was further conducted to examine the frequency effects on changes in hearing levels. Comparisons were also made between the initial diagnosis results and the first reliable behavioural audiometric results to determine the proportion and extent of hearing changes. Further explorations on the differences in clinical characteristics were performed by comparing children with and without hearing deterioration using Fisher's exact test or Chi-Square analysis, as appropriate. All analyses used two-tailed tests, with statistical significance set at *p* < 0.05. The statistical analysis was performed using IBM SPSS Statistics for Windows v.29 (43).

## 3. Results

### 3.1. Demographic characteristics

Table 1 provides descriptive statistics of the demographic characteristics at diagnosis of the sample. The etiology records were extracted from clinical reports or provided diagnosis reports available at Hearing Australia and were carefully reviewed by experienced research audiologist. Among a total of the 91 children, etiology was known for 35 children (38%), and the remaining 56 (62%) children had no reported etiology. Of the 35 children with known etiology, 10 (11%) of them had absent or abnormal auditory nerves, 9 (10%) were born with atresia and/or microtia, 8 (9%) had Cytomegalovirus (CMV), 4 (4%) had inner ear anomalies (enlarged vestibular aqueduct syndrome), 4 (4%) had syndromic hearing loss (1 Down's syndrome, 1 Goldenhar syndrome, 1 Noonan's syndrome, 1 Global developmental delay). Among the 56 children without a reported etiology for their hearing loss, 4 individuals were suspected to have a genetic basis, as one of their parents had a history of hearing loss, suggesting a potential hereditary component to their hearing loss. The distribution of degree of hearing loss at diagnosis in the sample showed that out of the 91 children, 1.1% had a high-frequency mild hearing loss, 16.5% had a mild hearing loss, 13.2% had a moderate hearing loss, 25.3% had a moderate to severe hearing loss, 15.4% had a severe hearing loss, and the remaining 28.6% had a profound hearing loss. The use of hearing aids was also recorded. By the time the children obtained their first reliable behavioural audiogram, 62 out of 91 children were fitted with hearing aids. Another 2 children were fitted with hearing aids within one month after obtaining the first behavioural audiogram. The remaining 27 children were not fitted with hearing aids at the time of the first behavioural audiogram.

**Table 1:** Demographic characteristics of children with congenital hearing loss (*N* = 91).

Characteristics	Children
Gender	
Male, *n* (%)	46 (50.5%)
Female, *n* (%)	45 (49.5%)
Birthweight, kilograms	
Mean (SD)	3.2 (0.65)
Median	3.3
Interquartile range (IQR)	2.9–3.6
Missing data, *n* (%)	18 (14%)
Gestation, weeks	
Mean (SD)	38.1 (2.8)
Median	39.0
IQR	37–40
Missing data, *n* (%)	16 (17.6%)
Age at diagnosis, months	
Mean (SD)	2.1 (1.2)
Median	1.7
IQR	1.2–2.6
Affected ear at diagnosis	
Left ear, *n* (%)	48 (52.7%)
Right ear, *n* (%)	43 (47.3%)
Etiology, *n* (%)	
Absent/abnormal auditory nerve	10 (11%)
Atresia/Microtia	9 (10%)
Cytomegalovirus (CMV)	8 (9%)
Large vestibular aqueduct syndrome (LVAS)	4 (4%)
Other syndromes	4 (4%)
No reported etiology	56 (62%)
Degree of hearing loss at diagnosis^a^, *n* (%)	
High frequency	1 (1.1%)
Mild [20–40 dB]	15 (16.5%)
Moderate [41–55 dB]	12 (13.2%)
Moderate to Severe [56–70 dB]	23 (25.3%)
Severe [71–90 dB]	14 (15.4%)
Profound (>90 dB)	26 (28.6%)
Hearing device fitting, *n* (%)	
Before the first reliable behavioural audiogram	62 (68.1%)
One month within the first reliable behavioural	2 (2.2%)
5 months after the first behavioural audiogram	5 (5.5%)
Not fitted by time of the study	22 (24.2%)

^a^ Degree of hearing loss was categorized as: high frequency loss only: ≥25 dB eHL at ≥2 frequencies above 2 kHz (23); mild hearing loss: 20–40 dB; moderate hearing loss: 41–55 dB; moderate severe hearing loss: 56–70 dB; severe hearing loss: 71–90 dB; profound hearing loss: >90 dB (23, 25).

### 3.2. Age when the first reliable behavioural audiogram is obtained

**Figure 1:**
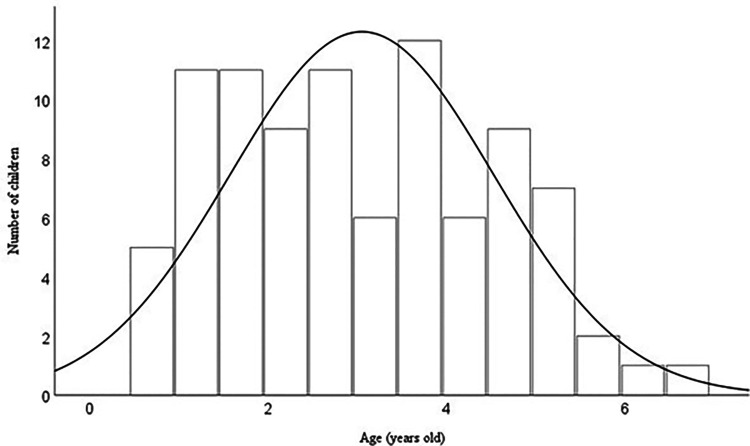
Histogram of age (years) at which the first reliable behavioural audiogram was obtained.

Figure 1 shows a histogram of the age at which the first reliable audiogram was obtained, with a mean age of 3.0 years (SD: 1.5; IQR: 1.8, 4.1). Although the mean age at diagnosis of the sample was 2.1 months old, it has been noted that about half of the children (47.3%) did not obtain their first reliable behavioural audiogram until after 3 years of age. A correlation analysis showed no significant relationship between the degree of hearing loss (3FA HL) and the age at which the behavioural audiometry was obtained (*p *> 0.05).

To explore the potential factors contributing to the delay in obtaining reliable behavioural audiograms, a multiple regression analysis was conducted. The dependent variable was the age at which the first reliable behavioural audiogram was obtained. The independent variables consisted of 4 clinical variables (presence or absence of reported etiology, degree of hearing loss at diagnosis, ongoing middle ear problems in the impaired ear, and hearing device fitting), as well as 3 additional factors (whether children comply or not with the testing, presence or absence of other faced challenges unrelated to children's behavioural issues, and family availability) (see details Section 2.2. “Factors affecting the age of obtaining reliable behavioural audiograms”). Regarding the hearing device fitting factor, it was categorized into two groups. One group included cases where a hearing aid was fitted before or around the time of obtaining the first reliable audiogram. The other group included cases where a hearing aid was fitted long after the first reliable audiogram or where no hearing devices were fitted at all.

The results in Table 2 revealed a significant regression model [*F*(7, 83) = 2.63, *p* = 0.017] with the full set of predictors accounted for 11.3% of total variance in the age of getting the first reliable audiogram. Among these predictors, children's behavioural issues (*β* = 12.9, *p* = 0.003) and the presence or absence of reported ongoing middle ear issues (*β* = −10.84, *p* = 0.04) demonstrated significant associations with the dependent variable. Specifically, children with reported behavioural issues were likely to be older when getting their first reliable audiogram. Conversely, those with reported ongoing middle ear issues tended to be younger when obtaining their first reliable audiogram. However, for the remaining predictors, there was insufficient evidence to demonstrate significant associations with the dependent variable.

**Table 2:** Effect size (unstandardized coefficient estimates Beta-values), 95% confidence intervals (95% CI), and significance levels (*p*-values) of predictor variables for age at first reliable behavioural audiogram (*N* = 91).

	Beta	95% CI	*p*-value
Presence or absence of reported etiology	1.70	(−5.69, 9.09)	0.65
Degree of hearing loss at diagnosis	−0.01	(−0.14, 0.13)	0.94
Ongoing middle ear problems	−10.72	(−20.76, −0.67)	0.04*
Hearing device fitting	−1.44	(−9.54, 6.67)	0.73
Presence or absence of children's behavioural issues	13.12	(4.94, 21.30)	0.002*
Presence or absence of other faced challenges	−4.37	(−19.69, 10.95)	0.58
Family availability	9.23	(−3.02, 21.48)	0.14
Adjusted *R*^2^	0.112		

* Depict significance at 0.05 probability level.

### 3.3. Change of hearing threshold levels

#### 3.3.1. Relationship between the hearing thresholds at diagnosis and the first reliable behavioural assessment across tested frequencies

**Figure 2:**
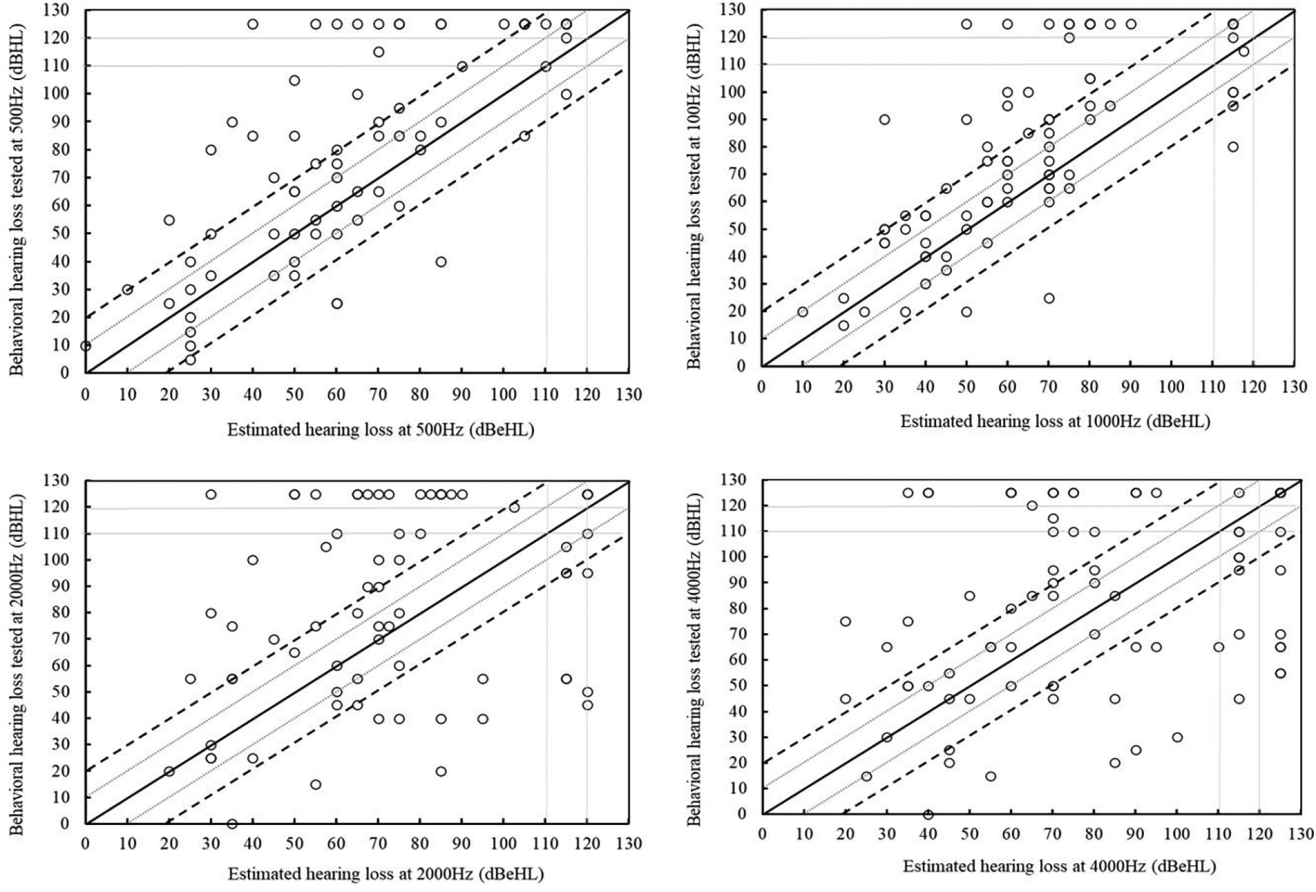
Scatter plots showing the estimated threshold at diagnosis (horizontal axes) against the hearing thresholds at the first reliable behavioural assessment (vertical axes) at 500, 1,000, 2,000, and 4,000 Hz. The solid diagonal line shows agreement. Points above the solid line indicate deterioration. The range between the two grey lines depicts +/− 10 dB, and that between the two dotted depicts +/− 20 dB.

Figure 2 illustrates the relationships between the baseline hearing thresholds of the impaired ear at diagnosis and the values measured at the first reliable behavioural audiogram across tested frequencies. The results depict significant correlations between the estimated hearing threshold (dB eHL) and the measured hearing thresholds (dB HL) from behavioural assessment at each tested frequency (*r* > 0.75, *p *< 0.001). The strongest positive correlation between the two measures was observed at 1 kHz [*r*(89) = 0.8, *p *< 0.001], while the lowest correlation was found at 500 Hz [*r*(74) = 0.75, *p *< 0.001] with greatest variability in hearing changes [mean difference of 14.3; SD: 25.0, IQR: (0, 21.3)]. In Figure 2, the data points above the solid line represent participants whose hearing thresholds were higher in the behavioural testing, indicating a deterioration, while points below the solid line represent an improvement in hearing threshold at the behavioural test.

A two-way ANOVA test was conducted to examine the effects of four tested frequencies (0.5, 1, 2, and 4 kHz) and five age ranges on the dependent variable of hearing level changes. The age ranges considered were under 2 years, 2–3 years, 3–4 years, 4–5 years, and over 5 years, which correspond to the age at which the first reliable behavioural audiogram was obtained. The results revealed a statistically significant difference in the overall changes in hearing thresholds among the four frequencies [*F*(3, 307) = 2.83, *p* = 0.039]. Post-hoc analysis indicated that the changes in hearing levels were significantly higher at 500 Hz compared to the other frequencies, followed by 1 kHz, 2 kHz, and the lowest changes were observed at 4 kHz. The main effect of age of getting the behavioural audiogram on the changes in hearing levels [*F*(4, 307) = 0.83, *p* = 0.51], and the interaction between frequency range and age [*F*(12, 307) = 0.45, *p* = 0.94] were not statistically significant.

Although there were generally high correlations between the hearing thresholds at two measured points, a considerable number of children exhibited either deterioration or improvement at each frequency. To explore this further, a detailed analysis of hearing level changes at tested frequencies was conducted (as shown in Table 3). The results indicated that majority of children experienced deterioration rather than improvement in the initial impaired ear at each frequency. For instance, at 1,000 Hz, 58 children (63.8%) had hearing deterioration of 10 dB or more, while 12 (13.2%) demonstrated hearing improvement of 10 dB or more. Overall, there were more instances of hearing changes (either deterioration or improvement) in the 500 and 1,000 Hz frequency ranges. Approximately 79% and 77% of ears showed a hearing change of 10 dB or more at 500 and 1,000 Hz, respectively. In contrast, less than 62.5% of ears experienced a hearing change at 2,000 Hz and 4,000 Hz.

**Table 3:** Number (%) of children demonstrating hearing changes and average hearing level change (mean ± SD) at tested frequencies.

		Frequency (Hz)			
		500	1,000	2,000	4,000
Available data points (*n*)		76	91	72	88
Number (%) of children demonstrating hearing change					
Deterioration	≥20 dB	30 (39.5%)	28 (30.8%)	18 (25.0%)	19 (21.6%)
	≥10 and <20 dB	16 (21.1%)	30 (33.0%)	13 (18.1%)	15 (17.0%)
	subtotal	46 (60.6%)	58 (63.8%)	31 (43.1%)	34 (38.6%)
Improvement	≥20 dB	5 (6.6%)	4 (4.4%)	5 (6.9%)	9 (10.2%)
	≥10 and <20 dB	9 (11.8%)	8 (8.8%)	9 (12.5%)	11 (12.5%)
	subtotal	14 (18.4%)	12 (13.2%)	14 (19.4%)	20 (22.7%)
Any hearing change (include deterioration or improvement)	≥20 dB	35 (46.1%)	32 (35.2%)	23 (31.9%)	28 (31.8)
	≥10 and <20 dB	25 (32.9%)	38 (41.8%)	22 (30.6%)	26 (29.5%)
	Total	60 (79%)	70 (77%)	45 (62.5%)	54 (61.4%)
Average change of hearing level in dB					
Deterioration	Mean (SD)	28.9 (19.4)	23.9 (16.9)	28.7 (18.4)	26.1 (16.6)
Improvement	Mean (SD)	18.9 (11.3)	18.8 (11.7)	18.9 (12.9)	22 (12.0)
	Difference *p-*value*	0.011	0.11	0.024	0.15

* Independent Samples *t*-tests were used to compare the average change of hearing level between the two deterioration and improvement groups at each frequency.

By analysing the averaged hearing level changes in children with either deteriorated or improved hearing thresholds, it was observed that although the mean values of deterioration or improvement of thresholds were similar across all tested frequencies, the degree of thresholds difference was in general higher for deterioration than for improvement in these children. For example, at 500 Hz, the mean deterioration in hearing thresholds was 28.9 dB, compared to the mean improvement of 18.9 dB. The statistical analysis with independent *t*-test indicated that these differences were statistically significant at 500 and 2,000 Hz [at 500 Hz, *t*(38) = 2.4, *p *= 0.01; at 2,000 Hz, *t*(35.2) = 2.0, *p *= 0.024].

#### 3.3.2. Examination of the proportion of children who had hearing loss changes

**Figure 3:**
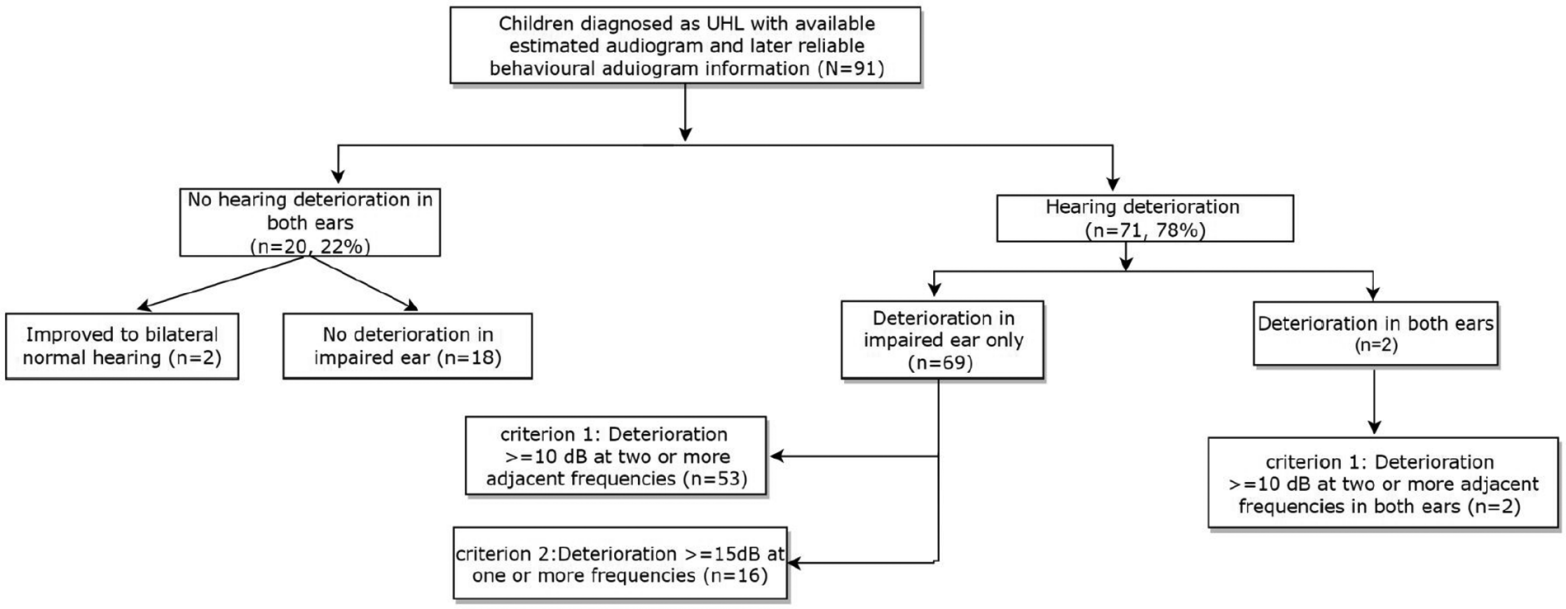
Flowchart of the number of children with and without hearing deterioration, including details on the extent of hearing deterioration based on definition 1 for progressive hearing loss.

Table 4 shows the proportions of hearing loss changes in the impaired ear. Using definition 1, approximately 78% of the 91 children (*n* = 71) experienced deterioration at the time of the first reliable behavioural audiogram. Among the 71 children with hearing deterioration, 34 of them demonstrated deterioration by 3 years of age. As shown in Figure 3, two children progressed from unilateral to bilateral hearing loss by 3 years of age. Out of the 69 children who had hearing deterioration in the impaired ear only, 53 of them had a decrease in 10 dB or greater at two or more adjacent frequencies between 0.5 and 4 kHz, and the other 16 children had a hearing decrease in 15 dB or greater at one octave frequency in the same frequency range. For the 20 children who had no hearing deterioration in both ears, two of them had behavioural thresholds within the normal hearing levels in both ears.

**Table 4:** Proportion of hearing loss changes in the impaired ear (*N* = 91).

Definition of progressive hearing loss		Number (%) of children with hearing loss changes in the present study		
		Deterioration (*n*, %)	No deterioration (*n*, %)	
Definition 1	criterion (1): a decrease in 10 dB or greater at two or more adjacent frequencies between 0.5 and 4 kHz, or criterion (2): a decrease in 15 dB or greater at one octave frequency in the same frequency range	71 (78.0%),[Including 55 (60.4%) children who met criterion 1; and an additional 16 (17.6%) children who met criterion 2]	20 (22.0%)	
		Proportion of children progressed to severe to profound loss 52/71 (73.2%)		
		Deterioration (*n*, %)	Stable^a^ (*n*, %)	Improvement^b^ (*n*, %)
Definition 2	a change of ≥20 dB in the three frequencies (500, 1,000, and 2,000 Hz) pure-tone average	24 (26.4%)	65 (71.4%)	2 (2.2%)
		Proportion of children progressed to severe to profound loss 22/24 (91.7%)		

^a^ Definition of stable: the three-frequency (500, 1,000, and 2,000 Hz) average hearing level (3FA HL) of the behavioral results is less than 20 dB difference (either increase or decrease) from the diagnostic results.

^b^ Definition of improvement: an increase of greater than or equal to 20 dB (i.e., ≥20 dB improvement) in the 3FA HL.

As shown in Table 4, when applying definition 2 (i.e., 3FAHL ≥ 20 dB), 26.4% of the children in the sample experienced hearing deteriorated by the time of the first reliable behavioural audiogram, while 2.2% showed improvement in the hearing levels. The majority of children (71.4%) had stable hearing between the two measurement points. Among those with hearing deterioration (*n* = 24), 23 demonstrated deterioration in the initially impaired ear, and one child was identified as having hearing deteriorated in both ears.

We also observed that a high proportion of children in our study developed severe to profound hearing loss from a milder degree of loss. Specifically, when applying definition 1, out of the total of 71 children who showed progressive hearing loss, 52 of them (73.2%) deteriorated to severe to profound hearing loss, compared to their initial hearing thresholds at the time of diagnosis. When using definition 2, 22 out of the 24 children, an overwhelming majority of them (92%) deteriorated to severe to profound hearing loss during behavioural assessments.

#### 3.3.3. Clinical characteristics of children who had changes in hearing loss

Table 5 showed the breakdown of clinical characteristics and etiology information for children with or without hearing deterioration based on the two different definitions. According to the frequency-specific definition of progressive hearing loss (Definition 1), children with varying degrees of hearing loss at diagnosis may exhibit hearing deterioration in at least one frequency. On the other hand, based on definition 2 (i.e., 3FAHL ≥ 20 dBHL), apart from the moderate (*n* = 12) and severe (*n* = 14) two groups, more than 70% of the children in any other degrees of hearing loss at diagnosis are likely to remain in the stable group. For example, among the 15 children diagnosed with mild hearing loss, 12 of them (80%) showed no hearing deterioration based on definition 2. This is because definition 2 may not capture further deterioration in certain frequencies. By excluding all children diagnosed with profound hearing loss, whose hearing loss may have already reached the limits of measured hearing thresholds, 60% (51 out of 85) would demonstrate deterioration according to definition 1, while 27.7% (18 out of 85) would show deterioration based on definition 2.

**Table 5:** Number (proportion) of children with or without hearing deterioration for clinical characteristics and etiology information based on the two definitions.

Characteristics	*N* = 91	Definition 1^a^		Definition 2^b^	
		Deterioration (*n* = 71)	No deterioration (*n* = 20)	Deterioration (*n* = 24)	No deterioration (*n* = 67)
Gender					
Female	45	37	8	14	31
Male	46	34	12	10	36
Hearing loss ear					
Left	48	36	12	15	33
Right	43	35	8	9	34
Degree of HL at diagnosis					
High frequency	1	1	0	0	1
Mild [20-40 dB]	15	11	4	3	12
Moderate [41–55 dB]	12	10	2	10	2
Moderate to Severe [56–70 dB]	23	18	5	7	16
Severe [71–90 dB]	14	11	3	6	8
Profound (>90 dB)	26	20	6	6	20
Hearing device^c^					
Fitted	62	49	13	18	44
Not fitted	29	22	7	6	23
Etiology					
Absent or abnormal auditory nerves	10	8	2	5	5
Atresia/Microtia	9	2	7	0	9
CMV	8	6	2	4	4
LVAS	4	4	0	2	2
Syndromic	4	2	2	0	4
No reported etiology	56	49	7	13	43

^a^ Definition 1: criterion (1): a decrease in 10 dB or greater at two or more adjacent frequencies between 0.5 and 4 kHz, or criterion (2): a decrease in 15 dB or greater at one octave frequency in the same frequency range.

^b^ Definition 2: a change of ≥20 dB in the three frequencies (500, 1,000, and 2,000 Hz) pure-tone average.

^c^ Hearing device fitted or not fitted before the first behavioural audiogram.

Furthermore, the deterioration rates were similar between the group of children fitted with hearing devices and the group without hearing devices, based on both definitions. According to Definition 1, 79% (49 out of 62) of the fitted group showed hearing deterioration, while 21% (13 out of 62) remained stable. In the not fitted group, 76% (22 out of 29) demonstrated deterioration, and 24% (7 out 29) remained stable. Similarly, based on Definition 2, the proportions were similar, with 79% of the fitted group experiencing deterioration, and 21% remaining stable. Among the not fitted group, 76% showed deterioration, and 24% remained stable.

Regarding the documented constellation of etiology, using Definition 1, 8 out of 10 children with absent or abnormal auditory nerves, 6 out of the 8 children with CMV, and all the children with LVAS demonstrated further hearing deterioration in at least one frequency. Among the 4 children with syndromic hearing loss, 2 experienced hearing deterioration, while the other 2 did not. The results by using definition 2 showed that the number of children whose documented constellation of etiology (including absent or abnormal auditory nerves, CMV, LVAS) were the same between the two groups of children with and without hearing deterioration. None of the 4 children with syndromic hearing loss showed hearing deterioration. Among the total of 9 children who were born with atresia and/or microtia, both definitions suggested a higher likelihood of stable hearing.

## 4. Discussion

The primary objective of this study was to report the clinical characteristics in a group of children with congenital unilateral hearing loss. We aimed to gather demographic information, determine the age at which the first reliable behavioural audiogram was obtained, and identify possible factors for any delays in acquiring the audiogram. Another important aspect of the present report was to directly compare the estimated hearing thresholds at the time of diagnosis with the first reliable behavioural audiogram, to investigate the hearing changes between these two measured points. This knowledge will contribute to the development of optimal audiological management, which will enable clinicians to promptly identify any hearing changes and make necessary adjustments to intervention strategies.

### 4.1. Demographic characteristics and etiology

The demographic characteristics of our sample demonstrate balanced representations of male and female children, as well as left and right of UHL. The equitable distributions suggest that UHL affects both genders and ear laterality without exhibiting any significant preference or potential biases. Among the 91 children included in our study, the etiology was known for 35 of them (38%). Absent/abnormal auditory nerve (*n* = 10) and ENT malformations (atresia or microtia) (*n* = 9) accounted for approximately half of the cases with known etiology. Among the 8 children with CMV, 2 were diagnosed with mild or moderate hearing loss, whilst the remaining 6 had severe to profound hearing loss. This finding aligns with previous reports indicating that CMV infection is associated with such hearing loss severity (36–38). Considering this significant impact, it underscores the importance of implementing CMV screening and genetic testing for children diagnosed with UHL. In addition, another 8 out of the 91 children (8%) in our sample had UHL associated with LVAS (*n* = 4) or other syndromes (*n* = 4). Among these children, the degree of hearing loss at diagnosis ranged from mild to profound, with a significantly higher percentage of moderate hearing loss observed in children with LVAS (3 out of 4 children with LVAS). Notably, 62% of the children in our sample had no reported etiology. Although this finding aligns with previous research reporting a high proportion of UHL cases with unknown etiology or no reported risk factors (23, 26, 34), the fact that a significant number of cases remain of unknown etiology presents a challenge for clinicians in understanding the underlying causes of UHL and developing targeted intervention strategies for these children.

### 4.2. Age at first reliable behavioural audiograms

Numerous published guidelines by various international organisations have outlined recommendations of early identification, assessment, and management of children with all forms of hearing loss, including that are UHL [e.g., (44–46)]. The results of this study revealed that 52.7% of the children (*n* = 48) identified with UHL through UNHS had their first reliable behavioural audiograms in the impaired ears by 3 years of age, despite a national pediatric clinical protocol recommending that ear-specific behavioural thresholds at all frequencies from 0.5 to 4 kHz should be obtained by 18 months of age for infants with hearing loss (47). This finding also contradicts the perspectives of clinical paediatric audiologists based on our online survey (see Footnote 1), where 64% of clinicians believed that a behavioural audiogram for individual ears could be reliably measured before 2 years of age, and 83% believed it could be obtained before 3 years of age for children with UHL. The potential reasons for this discrepancy could be related to clinicians' opinions on the clinical management of individual children and prioritizing of other clinical needs or activities for children with certain characteristics. For instance, in the case of children with severe to profound loss or absence of auditory nerve in one ear, clinicians tend to focus on the hearing in the normal ear and middle ear status. Similarly, this also applies to children with unilateral microtia or atresia as the hearing thresholds in the affected ear would not impact device settings.

The regression analysis from this report indicated the effects of degree of hearing loss, etiology, or hearing device fitting were not significant factors influencing the age at which behavioural audiograms were obtained. This differs from the aspects being identified as potential influencing factors by clinicians in an online survey, as detailed in Section 2.2, “Factors affecting the age of obtaining reliable behavioural audiograms”. Instead, younger ages of obtaining the first reliable behavioural audiogram were significantly associated with better child's behaviour and the presence of ongoing middle ear issues in the impaired ear.

Children with middle ear issues tended to have more appointments with clinicians, providing them with more opportunities to have hearing tests at early age. However, ongoing middle ear pathology might also delay the behavioural testing due to the potential for inconsistent test results caused by fluctuations in hearing levels or the need for a recovery period after medical treatment for middle ear conditions. It should be noted that only audiograms taken outside periods of temporary middle ear dysfunction, and meeting other criteria as outlined in Section 2.2, were considered as reliable for further analysis in this study. This exclusion may underestimate the age at which reliable audiograms could be obtained, as temporary middle ear dysfunction may not always impact hearing thresholds or diagnostic classifications. In addition, the factors identified in this report only accounted for 11% of the total variance in age at behavioural audiometry. Hence, additional research is needed to identify and gain a deeper understanding of the various factors contributing to delays in obtaining reliable behavioural audiograms. In particular, the attitudes of clinicians and parents or caregivers towards management of UHL in young children should be investigated (48–52). Understanding these perspectives will provide insights into how decision is made regarding audiological follow-up appointments, the prioritization of clinical activities, and the perceived importance of complete ear-specific audiograms. This information can help develop targeted strategies to address challenges and ensure children with UHL receive timely and comprehensive hearing rehabilitation.

### 4.3. Change in hearing threshold levels

Children's hearing thresholds may experience changes over time, which could be partially due to changes in ear canal acoustics (53, 54). However, the magnitude of such changes because of coupling changes is much smaller than what has been observed in the current findings (42). Past studies underscore the risk of hearing deterioration in children with any degree or type of hearing loss, and the deterioration can occur in one or both ears and its severity can range from mild to severe (16, 22, 29). Moreover, these changes can impact a child's perception of sounds across frequencies, thus affecting their language and learning development and overall functional performance (55–57). Hence, it is crucial to obtain reliable behavioural audiograms at an early age to identify and monitor any hearing changes.

This study indicates that changes in hearing levels were observed in each testing frequency. These changes could be classified as deterioration to improvement and categorised into a significant change (≥20 dB) or minor change (≥10 dB but <20 dB) at each frequency. The mean hearing deterioration for frequencies at 0.5, 1, 2, and 4 kHz ranged from 23.9 to 28.9 dB, while the mean hearing improvement ranged from 18.8 to 22 dB across the same frequencies (see Table 3). The analysis of the percentage of children experiencing hearing deterioration and improvement shows a greater proportion of deterioration rather than improvement at each frequency. Moreover, there are more instances of hearing changes in the 500 Hz and 1,000 Hz frequency ranges compared to the 2,000 Hz and 4,000 Hz ranges. These results are not consistent with a recent report by Fitzpatrick et al. (23) that showed similar hearing changes across frequencies. Allocating sufficient time and resources to obtain behavioural hearing thresholds at all frequencies is crucial. The present results suggest the importance of prioritizing the acquisition of low-frequency thresholds, considering the significance of low-frequency hearing for speech understanding, especially in noisy environments (58).

The current study found that 71 (78%) children diagnosed with UHL at birth experienced hearing deterioration between the diagnosis and first behavioural audiogram at one or more frequencies between 0.5 and 4 kHz (definition 1, see Table 4). Remarkably, among these 71 children, a high proportion of them (73.2%) developed severe to profound hearing loss (see Table 4). Previous studies using the same definition reported a deterioration rate of about 37%–47% of children with UHL when comparing the diagnostic audiogram with the most recent audiometric assessment (22, 23, 26). One possible explanation for the difference in proportions between the current study and previous reports may be attributed to the characteristics of the samples across studies. In Fitzpatrick et al.'s studies (23, 26), only about half of their samples were congenital UHL (i.e., 53.7% were congenital UHL in the 2023 study; 47.2% had congenital UHL in the 2017 study). The recent study (2023) reported that among the congenital UHL group (*n* = 95), 51 (54%) showed progressive HL. Another possible explanation may be that both Fitzpatrick studies compared the diagnostic audiogram with the most recent audiometric assessment. The mean length of time between assessments for children with progressive hearing loss was 64.3 and 50.3 months in the 2023 and 2017 studies, respectively. In contrast, our study only included children with congenital UHL, and we compared the results by examining the initial estimated hearing thresholds with the first behavioural audiogram. This approach allowed us to provide timely support after UNHS, for early identification and intervention in children with hearing deterioration. Using averaged three frequency thresholds (i.e., definition 2) to define deterioration, we only identified 26.4% of children in the sample as having hearing deterioration. This suggests that adopting averaged thresholds to define deterioration may underestimate the proportion of children experiencing hearing changes, which could have direct impact on management decisions.

This study also revealed that among the 48 children who had their first reliable behavioural audiogram before 3 years of age, 34 of them (71%) experienced progression of hearing loss in at least one frequency. This finding also emphasises the importance of monitoring hearing thresholds at all audiometric frequencies after diagnosis through newborn hearing screening. Early identification of deterioration has direct implications for considerations such as hearing device fitting and adjustments, evaluations of the impact of hearing loss on a child's development and determining appropriate intervention.

### 4.4. Strengths, limitations and future directions

One strength of this study is that it only includes children diagnosed with congenital UHL via UNHS, and it conducts a direct comparison between the initial estimated hearing thresholds from electrophysiological measures with the first reliable behavioural audiogram. Both of these methods have been recognised as the gold standard tests for threshold estimation for young infants (59). In this way, clinicians can promptly identify any hearing changes and adjust intervention plans as soon as needed. Another strength of this study is that the behavioural assessment procedures adhere to a nationally standardised clinical protocol implemented by Hearing Australia, a government-funded hearing service organization. By applying the same clinical protocol and using standard training and equipment, this ensures a relatively consistent and reliable approach in conducting the assessments. The diagnostic protocols are also well-established in audiological diagnostic hospitals/centres across Australia. This ensures the methodological rigour and quality of the data collected. To strive for an early intervention goal of 1-2-3 (i.e., hearing screening by one month of age, audiologic diagnosis by two months of age, and enrolment in early intervention by three months of age), our findings highlight the challenges in obtaining timely behavioural audiograms for children with UHL. The observed delays suggest that additional strategies and resources may be needed to meet the desired early intervention goal.

The current study has some limitations that should be considered. Firstly, the current data were drawn from participants in a research study, which may restrict the generalizability of the results to the general population. Secondly, the behavioural audiogram is obtained retrospectively via the Hearing Australia database, which means that certain factors that could influence the test results, such as children's behavioural issues, family's engagement, resources or other challenges during the appointment, might not be recorded in the case notes. This could affect the analysis of the potential factors contributing to the delay in obtaining reliable behavioural audiograms. Additionally, the low variance explained by our regression model indicates that there may be other factors affecting the age at which children obtain their first reliable behavioural audiogram that were not considered in our analysis. Future research could explore potential factors, such as parental and clinician attitudes towards UHL, as well as the frequency of attended appointments. These investigations may inform clinical practice and guide early intervention strategies. Thirdly, identifying potential etiology causes of progressive hearing loss in children with UHL is crucial for clinicians to develop targeted interventions. However, a significant challenge arises from the unknown etiology of many cases in our sample, making the clinical characteristics and risks associated with hearing progression in children with UHL still inconclusive due to the lack of specific information. Close collaboration with ENT specialists may be beneficial in addressing this challenge, as it could facilitate a more efficient acquisition of etiological information. Further research is necessary to investigate risk factors associated with hearing progression in this population. Lastly, the age of the first reliable behavioural audiogram depends on the child and family's availability to attend audiological appointments. As a result, the actual age of hearing deterioration may be even younger than what was reported, but the identification of hearing changes would rely on when the behavioural test was conducted. Additionally, this report does not include follow-up behavioural audiograms after the first reliable audiogram. Future investigations will examine the comparison between the first and subsequent reliable behavioural thresholds to capture the hearing changes in children with UHL during early life.

## 5. Conclusion

The findings from this study contribute to the understanding of the demographic, audiological, and etiological characteristics of children with UHL, highlighting the importance of early monitoring of hearing changes and factors that influence the age at which reliable behavioural audiograms are obtained. By gaining a better understanding of this information and its implications for children's developmental outcomes, clinicians and researchers can strive to optimize early clinical management strategies for children with UHL. Overall, the results indicate that closely monitoring hearing loss after initial diagnosis is essential to ensure optimal interventions are implemented at the earliest age for this target group of children.

## Data Availability

The datasets presented in this article are not readily available because the datasets are not available outside of the research team as per Hearing Australia Ethics approvals. Requests to access the datasets should be directed to the corresponding author (Vicky W. Zhang, Vicky.Zhang@nal.gov.au) and Hearing Australia Human Research Ethics Committee Secretary (Phillip Nakad, Phillip.Nakad@nal.gov.au).

## Footnotes

^1^The survey consisted of two main questions: (A) what is the clinician's perspective regarding the time of getting reliable behavioural results for child's individual ear. Audiologists were instructed to select a response from a list of 5 responses including: (1) <1-year-old; (2) 1–1.5 years old; (3) 1.5–2 years old; (4) 2–3 years old; and (5) >3-year-old. (B) what are the most relevant reasons with regards to the potential delays in obtaining the behavioural audiogram. Audiologists were asked to select the top 3 most relevant reasons including (1) Appointment time; (2) Equipment resources (e.g., equipment setup, booth availability, testing materials); (3) Staff resources; (4) Child's behaviours/compliance; (5) Degree of hearing loss; (6) Type of hearing loss; (7) Caregivers’ attitude on UHL; (8) etiology; (9) Child's device fitting status; (10) Others. In addition to the closed response questions, audiologists also had the opportunity to provide comments and specifications on each selected option and additional comments or insights on these questions. The results subsequently used for the following statistical analyses and discussion on the reasons of delaying in getting behavioural audiogram of this report.
